# Robots in Healthcare? What Patients Say

**DOI:** 10.3390/ijerph18189933

**Published:** 2021-09-21

**Authors:** Núria Vallès-Peris, Oriol Barat-Auleda, Miquel Domènech

**Affiliations:** Department of Social Psychology, Universitat Autònoma de Barcelona, Campus de la UAB, 08193 Bellaterra, Spain; oriol.barat@uab.cat (O.B.-A.); miquel.domenech@uab.cat (M.D.)

**Keywords:** artificial intelligence, robots, care crisis, patients’ perspectives, interpretative repertoires, science and technology studies, ethics of care, COVID-19

## Abstract

In this paper, we analyse patients’ perspectives on the introduction of artificial intelligence (AI) and robotic systems in healthcare. Based on citizens’ experiences when hospitalised for COVID-19, we explore how the opinions and concerns regarding healthcare automation could not be disassociated from a context of high pressure on the health system and lack of resources, and a political discourse on AI and robotics; a situation intensified by the pandemic. Thus, through the analysis of a set of interviews, a series of issues are identified that revolve around the following: the empirical effects of imagined robots, the vivid experience of citizens with the care crisis, the discomfort of the ineffective, the virtualised care assemblages, the human-based face-to-face relationships, and the automatisation of healthcare tasks. In light of these results, we show the variability in patients’ perspectives on AI and robotic systems and explain it by distinguishing two interpretive repertoires that account for different views and opinions: a well-being repertoire and a responsibility repertoire. Both interpretative repertoires are relevant in order to grasp the complexity of citizens’ approaches to automatisation of healthcare. Attending to both allows us to move beyond the dominant (political) discourse of technology markets as the only way to respond to healthcare challenges. Thus, we can analyse and integrate patients’ perspectives to develop AI and robotic systems in healthcare to serve citizens’ needs and collective well-being.

## 1. Introduction

In recent years, healthcare systems around the world have been struggling with rising costs and seeking to improve their results, clinically, economically, and in terms of the general well-being of the population’s health [1]. Accompanying these challenges, an heterogenous number of technologies are associated with the promise of an improved public sector with digital services to support patients as well as facilitate the cost-effectiveness of the system and improve working environments for healthcare and social care professionals [2]. This process has been led by significant strides in the adoption and proliferation of automation spurred by technological advances in computing, sensing, networking, and communications [3]. Recent breakthroughs in artificial intelligence (AI) and machine learning (ML), which are considered the most important general-purpose technologies of our times [4], have broadened the scope of automation beyond mechanised labour and industrial robotics to knowledge, care work, and service activities [3,5].

In this context, robots have been progressively introduced into hospitals and other healthcare environments [6,7,8,9], because of the convergence of the techniques of Human–Robot Collaboration and the science of AI. Moreover, advances in ML have produced significant promises in the possibility that machines perform activities that were traditionally exclusively human, activities that include abstract problem-solving, perceptual recognition, social interaction, or natural language use [10]. In this new era of robotics—known as New Robotics [11]—machines are expected to not only perform repetitive, routine tasks in predictable environments, but are being deployed to collaborate with humans in a wide range of activities in noncontrolled environments [3,5]. These technological developments are accompanied by a narrative in the media and academic literature that argues that robots represent the promise to solve what is known as the ‘care crisis’: demographic changes in high-income countries are increasing the need for healthcare services, while the number of people to provide them has been reduced [12,13]. Healthcare robots, like other digital technologies, are launched as an important measure to meet this challenge [14], proposed as a solution to mitigate the shortage of healthcare workers and resources [15], and to enhance the autonomy of elderly persons, among many others [7,16,17]. In the face of this process, innovation and efficiency have long been claimed as ways to solve this situation [18], along with other types of proposals, such as privatisation and commoditisation of certain health services, presented as the answer to some of the problems of inefficient and unresponsive public health systems [19].

Although automation has a long-standing impact on employment, productivity, and the economic performance of companies and nations, the introduction of AI systems and robotisation in healthcare environments has an unprecedented impact on the automation of care and healthcare. Thus, in addition to the technological challenges involved in the development of such technologies, they also imply new ethical and social challenges ‘to ensure that the tremendous potential of automation and AI are harnessed to not just drive economic benefits but to also promote human and societal wellbeing’ [3] (p. 45).

Motivated by these considerations, in this article we analyse the opinions, values, and concerns of patients concerning AI and robotic systems, based on the study of their care experience when hospitalised. With this objective in mind, we conducted a qualitative study based on a set of interviews with people hospitalised for COVID-19 infection in Catalonia (Spain). In the following section of this introduction, we explain the relevance of studying the perspectives of patients hospitalised during the global pandemic caused by COVID-19, as well as some of the conceptual elements underlying the analysis. In the next chapters, we present the methodology of the study, the results of the interviews’ analysis organised around the main themes identified in the interviews, the discussion of the results through the theoretical interpretation of the results using the notion of the interpretative repertoires, and finally, we summarise, briefly, the main conclusions.

### 1.1. Robotics and COVID-19

The patterns of caring and attention required to respond to the COVID-19 pandemic are unlike any that high-income societies had become familiar with in recent decades [18]. Given the unprecedented health impact of COVID-19, governments around the world were forced to respond to the disease with limited information by implementing unprecedented policies designed to reduce the rate of spread of the infection. During the first year of COVID-19, the fight against the pandemic was arguably being conducted through the implementation of nonpharmaceutical interventions designed to curb infection rates. In general, these measures aimed to reduce transmission of the virus by reducing person-to-person contact, thereby slowing the spread of COVID-19 to a manageable rate that allowed for the care of cases by the national health systems [20]. In this context, expectations and development of AI and robots received a new impetus, due to their ability to be used to reduce physical contact between people and thus, prevent the spread of the virus [21].

The clinical care domain has the second largest set of uses for robotics (after public safety) in the context of COVID-19, generally in large hospitals or affiliated clinics, when a reliable wireless network and information technology support are in place [22]. Four major types of applications of robots are identified for use in hospitals to manage the pandemic situation [22,23]: (1) healthcare telepresence, for communicating without contact—which includes the use of teleoperation by doctors and nurses to interact with patients for diagnosis and treatment; (2) disinfection of the hospital or clinic—using ultraviolet light to perform a gross disinfection followed by a human wiping down of surfaces likely to have been missed; (3) prescription and meal dispensing, transporting materials and supplies—whereby carts navigate autonomously through a hospital; (4) telepresence robots—which involve processing the intake of patients and handling families, protecting the receptionists and clerks, enabling families to remotely visit patients, and automating inventory management for a hospital floor.

However, the global emergency has exacerbated some dynamics previously existent in healthcare in relation to AI and robotics: the saturation of healthcare systems as the justification, the technological solutionism, and the scarce critical public debate.

(a) *The saturation of healthcare systems as the justification.* In many high-income countries, the governance or management [18] of healthcare systems cannot be interpreted without introducing the notion of the so-called ‘care crisis’: healthcare systems face substantial challenges due to an increase of elderly people, a boost in the need for healthcare services, and fewer people to provide and finance the services [14]. In this context, in recent decades, it has been expected that the marketplace will address this issue [18]. To the already existing increasing costs, worsening outcomes, and healthcare systems’ oversaturation [1], an oversaturation of healthcare services caused by the pandemic has been added. In this scenario, AI robots are posited as a support for healthcare systems in managing the high demand for assistance caused by the infection, reducing patient care workload, strenuous/repetitive manual tasks, and managing relations between patients and healthcare systems [22]. In relation to patients’ benefits, care robots are presented for their potential to address physical isolation, providing continued social interactions and adherence to treatment regimens without fear of spreading disease, as well as providing remote access to assist during quarantines [24,25,26]

(b) *The technological solutionism*. Next to the narrative of AI as a solution to care pressures, there are plenty of promises around AI and ML in medical settings: early diagnosis by using image analysis in radiology, pathology, and dermatology, with diagnostic speed and excelling accuracy; personalised treatments that optimise the care trajectory of chronic illness; precision therapies for complex diseases; reduction of medical errors, or improvement of subject enrolment in clinical trials [24]. However, there is very little validation with data and evidence on what tasks such innovations can perform, how they can help medical professionals, or how predictions of clinical outcomes are useful for healthcare systems [1]. This promising character of robotics and AI does not stem from a belief that all healthcare needs will soon be attended to by ‘robot doctors’ [25]. Instead, the argument is based on the classic definition of AI as an umbrella term for a range of techniques that use machines to do tasks in a way that would be considered intelligent if performed by a human. As it is known, medical and healthcare problems have multiple causes, which need to be addressed from multiple angles. However, policymakers, clinical entrepreneurs, and informaticists increasingly argue that a key part of the solution will be AI, in particular ML [26]. This is what is known as technological solutionism, the belief that complex problems can be solved by technological intervention alone [27]. This trend has been reinforced during the pandemic: lacking protective material resources, the human capacity for contact tracing, or understanding of the disease, policymakers in higher-income countries turned to technology for a miracle. The technology sector responded with history’s most extensive hackathon, illuminating the mutual shaping of technology and public health policy [28] (p. 210).

(c) *Scarce public critical debate.* In addition to the fact that participation of patients in healthcare has been associated with improved treatment outcomes [29], the involvement of stakeholders in technological design positively influences a wide range of domains, aligning technological development with social needs and identifying those issues that raise ethical concerns or could have detrimental side effects [30]. Taking this seriously seems quite relevant to analysing citizens’ perspectives on AI and robots in healthcare, thus guaranteeing that such artefacts will be aligned with current societal challenges, ensuring ethical acceptability, social desirability, and sustainability [31] Although various studies have analysed public attitudes towards the use of robotics (on this issue, see the review by Papadopoulos et al. [32]), the attitudes of the various stakeholders who are directly involved in care have not been a priority of research. In spite of this situation, however, there are some studies that have been carried out on the views and attitudes of nurses and other health and social care workers regarding the use of social robots in health and social care [32]. However, the evidence related to the thoughts, priorities, fears, and hopes of patients themselves is scarce. One small exception to this tendency being the field of gerontology, where there is a relatively long tradition in the introduction of companion robots and socially assistive robots [7,33]. During the pandemic, the development of such artefacts and the claims about their benefits have been much more intense than the public debate and reflection on their social and ethical impact.

For these reasons, we take the situation provoked by the COVID-19 as a paradigmatic case that intensifies previous dynamics in relation to robotics and AI systems in healthcare. The study of patients’ experiences of care at such a critical moment can help us to identify the opinions and values relevant for citizenship in healthcare’s automation.

### 1.2. The Relational Approach to Patients’ Experiences

As proposed by Numerato et al. [34], we use the notions of citizens and patients interchangeably, given that patients as citizens are not only limited to their relationship with health or disease, but in relation with healthcare as a whole system that also encompasses carers and relatives, as well as neoliberal regimes that govern healthcare systems. From this idea, we approach patients’ perspectives on AI and robotics from the framework of science and technology studies (STS). From an STS approach, it is assumed that a specific technology is not just an artefact, but a network of devices, processes, and actors that become interrelated in an assembly [35]. Any technological innovation is a conglomerate of material, social, and semiotic relations in which technical, scientific, political, economic, social, or ethical considerations are intimately related in a heterogenous network [35]. This idea highlights the impossibility of separating the definition of technical problems from the socio-economic framework to which it is associated [36]. Based on this approach, the ontology of AI and robotics is relational, the AI system is not the point of departure, but the focus is on the framework of relations in which it participates [37].

From this approach, patient experience is understood as a relational achievement that involves the interplay of people, places, organisations, procedures, policies, artefacts, etc. [38]. This relational character is too often overlooked in healthcare ethics and healthcare research literature [39]. Patient care experiences, as technologies, are not as entities pre-existing to the relations that shape them in space and time, but as practices done in and through interactions [38] of people who, with the help of processes, protocols, routines, or machines, act to achieve good care [40]. In the same manner, these interactions occur in concrete healthcare systems, crossed by organisational forms, power structures, values, symbolic elements, etc. Emphasising the contextual nature of technological artefacts, Feenberg [41] proposes the instrumentalisation thesis. When AI systems are introduced into a healthcare setting, what they are and the mediations they enable depend on their use in a given environment. In that environment, the conditions of possibility of their use are configured, which may be different from those originally intended and those that were contemplated in their design [41]. From this idea, it is especially relevant to introduce patients’ perspectives in relation to health automation processes. The design of a technology does not determine society or the mediations it configures, because these mediations are shaped by factors both of the artefact and its environment of use [42]. Patients’ views and opinions about automation in healthcare, as well as its practices and experiences, configure how AI and robotic systems mediate in healthcare relations, as the functional possibilities of those technological systems and the values and organisational context where they are introduced configure patients’ views, practices, and experiences.

## 2. Materials and Methods

The presented study involves empirical qualitative research, based on a set of interviews of patients hospitalised with COVID-19 in Catalonia (Spain). We carried out 13 in-depth interviews with patients with COVID-19 in different Catalan hospitals during the first and second waves (in Catalonia, the first wave was registered from March to June 2020, and the second from October to December 2020 [43]). Interviews were conducted face to face using online platforms, lasted approximately one hour and a half, and the interviewees were aged between 58 and 83 years; seven were female and six were male, and all had a structured family environment (therefore, some of the dynamics identified in this study should also be complemented by similar studies with patients without family support, an issue that, as will be shown throughout the paper, is particularly relevant in shaping the experience of hospitalisation).

The interviews were semistructured, meaning that the interviewee and the interviewer had considerable freedom to direct the course of the interview, based on a script divided into three blocks: the first block asked respondents about the process of becoming ill with COVID-19 and being hospitalised. In this block, questions were asked about the detection and monitoring of the disease before hospitalisation, the severity of the case, the days of admission (in the hospital and also in the hotels set up later to follow quarantines), whether or not there had been admission to the intensive care unit, the relationship with the hospital’s medical and healthcare staff, the relationship with the other patients, the relationship with relatives, etc. The second block focused on the feelings about and the evaluations of the hospitalisation experience: what were their needs, what made them feel good, what did not make them feel good, what they valued most during hospitalisation, etc. Finally, in the third block, they were asked about specific tasks in the hospital: how these tasks were organised during their hospitalisation and whether they considered that these tasks could be carried out by a robot and whether this was desirable. The tasks they were asked about were as follows: carrying food and giving it, carrying medication, cleaning the room, measuring vital signs, getting out of bed, carrying out tests, assisting in displacements and movement, communicating results, relations with other patients, relations with family and friends, and entertainment activities.

Interviews were recorded and transcribed, and subsequently, a thematic analysis of them was carried out [44]. For the analysis, an inductive method was used to identify topics and subtopics, based on familiarisation with the data—reading the interviews over and over again, looking for topics, reviewing potential topics, and defining and naming new topics. A semantic analysis of the interviewees was performed—that is, what the interviewees said, combined with a latent analysis, interpreting in line with the theoretical framework what the interviewees wanted to say or express.

## 3. Results

The results of the interviews are organised around the main issues identified during a thematic analysis of them. Six main issues or themes articulate the results: the empirical effects of imagined robots, citizens’ vivid experiences with the care crisis, the discomfort of the ineffective, virtualised care assemblages, human-based face-to-face relationships, and the automatisation of healthcare tasks.

### 3.1. The Empirical Effects of Imagined Robots

Although we did not find any systematic analysis of the introduction of robotics in Catalan hospitals to fight COVID-19, nor the perspectives on them of the different actors, from the analysis of the media over the time comprising the first and second wage, the media reported the following use of robots in Catalan hospitals, by type of application: healthcare telepresence: one hospital; disinfection of the hospital using UVC light: five hospitals; prescription and meal dispensing, transporting materials and supplies: two hospitals; telepresence robots: zero hospitals.

However, in contrast, none of the patients interviewed had been interacting with these robots that were mentioned in the academic literature or the media. It must be noted, moreover, that some interviewed patients had been hospitalised in hospitals that were reported in the media as having introduced robots to fight COVID-19, as well as in others of the most pioneering hospitals in Catalonia. Without wishing to contradict the media or the academic literature, as the aim and methodology of our study was not to conduct a systematic and comprehensive analysis of the introduction of robots in healthcare during the pandemic, this issue is not anecdotal of citizens’ perspectives on robotics in healthcare. This phenomenon has been reported in classical studies on the ethical and social discussions on robotics, in which the prospective use of such artefacts operates as a chimerical idea around experimental projects [45].

Undoubtedly, not having interacted with any robot, and not being aware of the daily use of robots in the hospitals where they were admitted, contributed to the range of discourses and arguments developed during the interviews. However, more relevant is that public discourses on the benefits of robotics for fighting COVID-19 had also produced effects and are an inseparable part of robotics’ development and citizens’ perspectives on them:

*Researcher [R]—Can you imagine a hospital where you would have the same care and you would have been treated in the same way, but full of robots and everything very mechanised?*

*Interviewee [I]—Yes, I can imagine it. I will not see it, but it is the future. When you are alone in an isolated room, what do you care? You can’t go out in the hallway and talk to people, you’re alone.*

*R—Do you think that’s a good thing?*

*I—Yes, it’s positive, but I don’t know if robots will ever do more than people. I don’t know what it would be like, but I can see it. I think humans take advantage of everything, even a pandemic [...]. It is the government’s fault, but in this context, doctors need more resources and more help. For example, now I am calling a thousand telephones to talk to a doctor, and they don’t answer me because they can’t give me enough, they need help and support. The other day, to order the Covid-19 vaccine, I had to do it through the Internet, because, at the medical centre, which is three minutes away, there was a huge queue. Perhaps all those people who have had to delay operations and treatments would not have had to do so if there were machines to help them.*

Woman, 72 years.

In patients’ narratives, although they have not interacted with them, robots are a real future. Robots are not a fallacy, but artefacts that shape their perspectives on care as patients in a health pandemic scenario. In order to conciliate this dual existence of robotics, that exist but do not exist, we address robotics as imagined entities with empirical effects. Defining the question in this way, patients’ care relations with nurses, physicians, their families, and with digital technologies are considered as the empirical framework for analysing ethical and social concerns about introducing robots in healthcare. In this way, we can analyse how patients thought about and constructed a narrative around robotics, based on their care experiences during hospitalisation.

### 3.2. The Citizens’ Vivid Experience of the Care Crisis

Patients’ hospitalisation during the first and second waves of COVID-19 was an exceptional experience, marked by overcrowding and lack of resources. The impact of this experience was particularly acute during the first hours of admission to hospital:

*The overcrowding of so many people in the corridor waiting for a diagnosis or taking a test or listening to people complaining or vomiting... I didn’t want to hear any of it. I remember that night very strongly. I also remember very strongly changing places, first in one room, then in another. When I was discharged, I realised that I had lost a shoe. I remember those situations very strongly afterwards.*

Woman, 68 years.

*I went into the hospital diagnosed with coronavirus, so my time in emergency was shorter than other people’s, I was there for about 12 h. The emergency experience was dramatic: the scene was like something out of a movie, doctors and nurses running around, people piled up, broken grandparents. I was in a cubicle and at one moment they took one person out and put another one in because he had died. The feeling was... very stressful.*

Man, 61 years.

*When I arrived at the hospital, I spent 5 h waiting in the corridor […]. The hospital waiting room is all screened off. Here, you are in the war. I thought “Here I have to survive any way I can”. You suffer from decomposition. All the people are sick. You cry. Forty beds, it’s war medicine. I don’t know the result of the nasal swab. I’m there from 7 p.m. to 3 a.m. Then, they take me to a place they call the multipurpose room […]. Two days in the multipurpose room.*

Woman, 67 years.

Patients’ experiences during COVID-19 cannot be understood in isolation, but are entangled in a series of dynamics that shape health systems, dynamics that existed prior to the pandemic. The practices that take place in the context of a social and health emergency have aggravated a series of already existing tensions. In advanced industrial societies, less care work has been done, while the ageing of the population means an increased need for care. This process is accompanied by a definition of citizenship that does not make provision for responsibilities to care for others, and advanced industrial societies have not rethought who is responsible for care [46]. This is what is commonly defined as the ‘care crisis’. Globally, healthcare systems are struggling with rising costs, while seeking to improve their outcomes, clinically, economically, and in terms of the general well-being of the population [19].

This situation has been intensely exacerbated by the global pandemic, in which health systems have been placed under increased pressure to deliver assistance, causing their oversaturation. The interviews narrate how, during the first and second waves of the pandemic, the impact of the ‘care crisis’ has been dramatically incarnated on the citizens’ bodies: the oversaturation, the overcrowding, the lack of beds and medical personnel… all take place in the hospital within the body of patients suffering decomposition and vomiting in a corridor with other patients packed in together, crying or without one shoe.

### 3.3. The Discomfort of the Ineffective

Throughout the hospitalisation process, what is associated with discomfort and an unsatisfactory experience of care and attention has to do with the irrational and disorganised, with that which does not produce the expected responses. This affects, as explained above, the process of admission to hospital, but also many other processes:

- Diagnostics: when the same test is performed more than once, for example, X-rays or PCRs (which were not yet widely used in Catalonia during the first and second waves), whether in primary care centres, emergency centres, and/or hospitals, because the data and results are not shared between the various entities that make up the health system.
- Pre- and post-hospital follow-up: when no recognisable pattern is identified in the telephone follow-up prior to admission (for example, when citizens reported a worsening of symptoms on the app created by the health department, when the calls received did not correspond to a greater or lesser severity of symptoms, when no clear indications exist to go to hospital, etc.) or after hospitalisation:

*What has hurt me is not having a follow-up once out of the hospital, the post-treatment. I have had to do it myself. I don’t know if I’m fine. What I was telling you about my leg or lungs or headaches… No one tells me anymore and I have to manage. I take my temperature, but this is not ok, there is no follow-up and this is the only thing I miss.*

Woman, 72 years.

- Communication of the hospital with relatives: given that patients could not have companions in the room, each hospital established a series of procedures for communicating the evolution of his/her state of health to the relatives. In some interviews, cases of confusion about the patient’s history are reported, whereby some relatives receive news of the patient’s serious deterioration, which is later denied. This type of misunderstanding, even if it happens only once, causes a lot of anger and a significant lack of confidence in the organisation of patient records and the functioning of the communication system.
- Organisation of hospital spaces and routines: the saturation of the hospital during the first and second waves of the pandemic affected the different services of care and assistance involved in the hospital (management of the over-occupation of patients, routines of cleaning and disinfection, types of food delivery, times of medical visits, etc.), making it difficult to predict care and daily routines during hospitalisation:

*What made me feel even worse was the hole I was in. At no time did I think I might die, nor did I doubt that I would be well cared for. There were no clear instructions. The nurses told me they would come three times a day and then they came twice. This could not be. Especially with the cleanliness. Are you coming to change the bedsheets every day or not? If you have a disease where cleanliness and disinfection are so necessary, this is important. They threatened me that I had to eat. I was in a place that left you stiff, with a small soul. I was very “fucked up”... I attribute it to being in that place.*

Man, 72 years.

As part of the same process of the care crisis, during recent decades, healthcare has been exposed to the principles of neoliberal governance, with different impacts according to country. Healthcare has been increasingly decentralised, and the delivery of different services has frequently been privatised and transferred to new non-public providers or public providers with private management [36]. Fragmentation of care (provided by different providers under new public management formulas [47]) is the setting in which the social and health crisis caused by COVID-19 occurred. When these forms of neoliberal governance have not worked as expected during the pandemic, effectively managing the fragmentation of care, patients suffered a series of experiences linked to discomfort. This discomfort is not only related to the ineffective functioning of the service, but the deception on an expected rational, organised, and foreseeable functioning.

### 3.4. Virtualised Care Assemblages

Throughout the COVID-19 crisis, as in most countries, in Spain, hospitalised patients were obliged to be quarantined and in isolation, which means that they could not be accompanied by family members or receive visitors. In all the interviews, together with a detailed explanation of the evolution of the disease, this fact was central to the account of the experience. However, it has not meant a lack of communication with the family, nor an affective distancing from them. The relations of patients with their families have been constant via mobile phones, a particularly positively valued issue:

*The doctor brought me my mobile phone... I spent the whole day chatting, talking nonsense and letting myself be loved.*

Woman, 59 years.

*I had the mobile phone, they didn’t take anything from me. I talked every day with my brother, with my nephews, with my husband, with my children, with my friends, that is... I spent the day talking on the phone, thank goodness, otherwise I’d be bored as a monkey.*

Woman, 63 years.

In the life of hospitalised patients during the day, the mobile phone plays a central role in the relations with their relatives and friends. The mobile phone was the mediating artefact that made possible the affective care and emotional support of the families with their relatives and vice versa. Through the mediation of the mobile phone, a virtual assemblage of care was organised among the patient and her/his family and friends. In some cases, this relationship was organised through a single person, with whom the patient made a set of calls or video calls each day, so as not to have to talk to all their relatives, friends, and acquaintances and explain their condition. In other cases, this relationship was established with different people, and during the day, the patient made multiple calls and videos with different people.

Caring infrastructures mediated by the mobile phone make it possible to care for hospitalised patients at a time when the public health system is not capable of guaranteeing to do so. This care does not refer only to the emotional support and accompaniment of the patient, it also involves the management of care by relatives, who establish new links with the hospital to provide their relatives with pyjamas, slippers, soap, specific medicines for chronic diseases, etc. In the same way, the virtual infrastructures created are also used by the patient to be attentive to the health status of their relatives, to reassure them about their situation; thus, enabling bidirectional care:

*The first two days my husband only spoke to me and of course I couldn’t tell him how I was doing medically. Then, he called the hospital and asked how it is possible that no doctor or nurse called him to report how I was doing. From that moment, every day they called him. At night we made video calls with the whole family, and I felt very safe at all times […]. Then my husband asked if he could send me more clothes, toothpaste… and they sent it to me. At all times I felt accompanied and connected.*

Woman, 72 years.

*What I valued most when I was in hospital was being able to communicate with my family. My wife also had covid, and even though she was not in hospital, this way I could know how she was doing.*

Man, 61 years.

### 3.5. Human-Based Face-to-Face Relationships

The routines and the relationship between patients and medical and nursing staff during hospitalisation were different from what citizens were used to (from their own previous experiences, or from their knowledge of the healthcare system). Visits by doctors and nurses were much less frequent than usual, interactions were generally of a very limited time, and all hospital staff with whom the patient interacted were wearing personal protective equipment, making face and voice recognition difficult:

*R—Does the humane treatment that you mention exist with the nurses?*

*I—Yes, yes, but not enough. You saw one and then another, and you didn’t know if it was the same one. There came a time when you got lost. Sometimes they didn’t speak, sometimes they did, you couldn’t see their face.... You didn’t see their face. But they were attentive. Not a relationship to be able to talk and talk. I guess they didn’t have time for it either, poor people. But the people were attentive, they treated me well and it was at the hardest time of the pandemic.*

Woman, 72 years.

However, despite being different, and that the relationship had no previous reference in the patients’ experiences (they had never been attended at a time of such pressure of care, nor by health personnel whom they literally could not see or recognise), none of those interviewed showed dissatisfaction with the relationship with hospital staff. Sometimes, when patients explained that there were few visits from doctors or nurses or that interactions were very short, they were immediately told that their situation was understood and that they were overwhelmed.

In this situation, the few face-to-face relationships with healthcare personnel that were not so brief were highly valued:

*R—Do you think you were well cared for?*

*I —Yes of course. When they came to pick me up, they gave me a farewell cordon, and the nurses applauded as I left the room. I applauded them too because I had to thank them for their efforts.*

Man, 83 years.

*When the nurses came to take my vitals, it was very pleasant. It was two or three minutes. Since you were alone, it was very nice. They were very stressed. To receive them, every day I shaved and dressed. This interaction was very positive, and I appreciated it.*

Man, 72 years.

Regardless of the task or healthcare associated with an interaction, patients establish a direct relationship between human interaction and good care. This relationship is experienced with affection and emotion and reported from a very positive and comforting memory.

### 3.6. Automatisation of Healthcare Tasks

In the final part of the interview, based on their accounts of care experiences during their hospitalisation, patients were asked whether a robot could participate in any of the care tasks performed while in hospital. However, we cannot establish any common guidelines on the type of robot that citizens would consider appropriate to introduce in a hospital, or on any of the possible tasks or functions to be automated (taking vital signs, delivering food, cleaning, accompanying, facilitating relationships with people outside the hospital, etc.).

In the pandemic context and in the face of the physical distance measures also imposed in hospitals, it is considered that interaction with humans, whatever the reason or objective of the interaction, is always valuable and desirable, especially in the most vulnerable situations:

*Maybe I would get cold if a robot took my constants, but I would be indifferent because I’m fine. But it’s different if a robot enters with a person who is ill and who sometimes needs a smile, or who needs... I think that it depends on what functions a robot can do… But when a nurse enters, she/he smiles at you or holds your hand or something... And addresses you four words... For those who are sick, who are really sick, that is very much appreciated.*

Woman, 63.

For respondents, care always involves human–human interactions, a kind of relationship that is governed by certain routines but that are not entirely predictable. Human beings’ relationships are conceptualised as opposed to the automated, a characteristic associated with robots:

*Of course, there is an issue here that I don’t know how we could fix. I think it’s the human treatment that helps you. When someone gives you an injection, just asking you if you are feeling better, you are better off. If she/he asks you how you are, it helps you. Or when you ask what your temperature is, sometimes they don’t want to tell you. You have to play a little bit, insist. Of course, with a robot I don’t know.... You are alone for many hours.*

Woman, 72 years.

However, even though patients intensely value their face-to-face relationships with hospital staff, from their experience of the oversaturation of the healthcare system and the lived experience of lack of care resources, robots in healthcare are accepted as a possible solution. The same interviewees, during the course of conversation, included different responses and arguments regarding the tasks that a robot could or could not do:

*Robots can do many things, but I think it’s very important to be cared for by a person, to be able to talk, that the patient can express what they feel. There are patients who don’t really have anyone else to talk to or who don’t have a mobile phone. Then, robots can do many things, but there are situations where a person is needed […]. A robot to help, help. Because if there is a robot that can do tasks where people are not needed, it makes the job easier, especially when everything is saturated as it was.*

Woman, 72 years.

Robot acceptance does not have to do with the tasks they can perform, their appearance, or type of functionalities. In citizen interviews, acceptance of robots is contextual. The robot is understood as one more node in the healthcare system, a system with strong pressure on care, a lack of resources, and from which efficiency is demanded. In this sense, although patients prefer to have interactions with humans for care tasks, the use of care robots is accepted as a responsibility for the proper functioning of the health system.

## 4. Discussion

### 4.1. Beyond Variability in Patients’ Perspectives

Throughout the interviews, we could not find a single pattern in terms of patients’ opinions on the introduction of robots, or about the functionalities and tasks that robots can perform. On the contrary, different and conflicting voices were mobilised when citizens connected their perspectives on automation with their care experiences when hospitalised.

In the different interviews, as well as in the course of the same interview, that which has to do with the introduction of robots, or the automation of different care tasks, was valued or accepted in different ways. On the one hand, patients clearly preferred all forms of interaction in the hospital related to healthcare to be with people. In these types of opinions, patients placed ‘care’ and ‘cure’ on the same level [48]: ‘when you are in pain and alone, the smile of a nurse cures you’. The recounting of this type of experience refers to a certain social consideration of care, which points to the results that caring achieves, as well as to the recognition and transcendence of the value of care and the ethics of care [49]. On the other hand, patients thought that it may be positive to introduce robots to perform some caregiving tasks or, at least, it is a thing that is going to happen. They not only referred to using these artefacts as assistance and support for healthcare staff, but also in delegating tasks and activities to the robot, replacing humans. However, throughout the interviews, we did not find specific tasks or activities that could be substituted by robots on which there was a consensus among the interviewees.

These types of results reinforce the findings of a number of different studies conducted over recent years, which show ambivalence regarding citizens’ opinions on the uses and acceptance of robots to perform healthcare activities [17,50,51,52]. However, in the face of this variability, the explanations may be different. In general, the literature on the subject resolves ambivalent patients’ perspectives by focusing attention on ‘the nature of care activities’: in order to answer the question as to when and how care robots should be used and how they should be designed, it is first necessary to reflect on what the different elements of care activities are, and on the different values embedded in different care activities. From this argument, it could be determined which care activities should be left to humans and which could be fulfilled with or by a robot [50,51,52,53]. From this type of approach, the aim is to specify as much as possible the types of healthcare robots and tasks, assuming that when fully specified, patients’ perspective ambivalence will disappear [17,51]. However, based on the results of the study, we propose an alternative explanation for the variability of citizens’ opinions and perspectives that does not revolve around the nature of the healthcare activity that could be done by the robot, but on the care conception mobilised by citizens. Thus, if we explain the variability of patients’ perspectives by appealing to a greater specification and breakdown of tasks, what we do is isolate a small part of care from the whole context that makes that practice a care relationship [5].

### 4.2. Well-Being Repertoire and Responsibility Repertoire

As Gilbert and Mulkay [54] propose in their groundbreaking text, *Opening Pandora’s Box*, there is no single way to account for context. Participants’ discourse is context-dependent, but their description of social action and opinions is potentially variable. The social world is a multiple reality in which different ways of explaining patterns of action and belief in technoscience coexist. Incompatible accounts and concerns about healthcare robots are sociologically significant, because, if we look at them closely, we can see that the patients are talking about different things. Citizens highly value their face-to-face relationships with others (with doctors, nurses, and other hospital workers) while hospitalised. Citizens directly establish a link between relationships with humans, well-being, and good care. If these relationships cannot be face to face, as is the case with their family and friends who, with distance measures and quarantines, cannot accompany them while they are in hospital, virtual relationships (via mobile phones) are equally valued. Furthermore, although they clearly prefer relationships with humans, they accept, as a possibility, that some interactions in hospital may be replaced by machines. Additionally, not only do they accept it, but it is even understood as desirable.

Our results suggest that, when talking about their experiences of care, patients utilise what we referred to as a ‘well-being repertoire’, in which opinions and views about robotics are seen as a threat to good care, because good care is directly associated with the presence of humans. This presence has a special value when it is face to face, skin to skin, but does not exclude other forms of virtual relationships using digital technological devices. From this well-being repertoire, the form of relationship with hospital staff is a caring relationship. Good care is then exclusively human care. From this interpretative repertoire, robots are a threat to what is considered good care, because robots lack the properties that are considered indispensable for a care relationship: robots do not smile, do not touch, do not empathise, etc.

In contrast, when talking about the health system, its procedures, queuing, forecasting of services, organisation of pandemic responses, resources, etc., patients relied upon a ‘responsibility repertoire’, in which actions that robots can perform and the possibility of automatisation of tasks are viewed as positive and desirable. The system that treats them for COVID-19 disease, the people caring for them, is overwhelmed. This situation is not something alien to them, but they have experienced it in person during their hospitalisation process.

Patients accept and positively value the automation of care because, in this way, they take care of their carers, stressing the need for reciprocity in robotics [55]. Healthcare personnel are overburdened with work, and physically interacting with them increases their risk of being infected with COVID-19 and becoming ill. Introducing a robot makes it easier for patients and carers not to interact, with the acceptance of robots representing the patients’ willingness to care for the carers, even if it means renouncing what they consider to be ‘good care’ associated with human contact. Moreover, the concept of good care assumes different meanings under COVID-19 circumstances: powerful and effective public health responses have been relatively more successful in responding to the pandemic threat [18]. Therefore, good care, in this context, implies efficiency, an issue that cannot be dissociated from the narrative that accompanies the introduction of technologies in healthcare services, inextricably associated with the values of efficacy, effectiveness, and efficiency [14].

This dynamic responds to the more political dimension of care, the ‘caring with’, that shows how citizens are involved in relationships of interdependence and mutual care [56]. Patients trust that, over time, they will be able to reciprocate the care they have received from their co-citizens, using robotic devices to improve the efficiency of the healthcare system, avoiding hospital staff’s work overload and contagion among doctors, nurses, and other hospital workers. Thus, ‘the responsibility repertoire’ is associated with individual and collective responsibility to facilitate the proper functioning of the system and the guarantee of health assistance in a context of a lack of resources. In other words, the responsibility repertoire is the assumed citizens’ accountability of caring for their carers and their own health.

### 4.3. Robotics and AI as a Political (Neoliberal) Response to Healthcare Problems

The identification of different interpretive repertoires allows us a complex approach to the opinions and perspectives that diverse stakeholders have on a scientific-technical issue. The identification of the well-being and the responsibility repertoires enables us, on the one hand, to comprehend apparently contradictory opinions and perspectives on healthcare automation and, on the other hand, to identify a number of issues that may make plausible the introduction of robotic and AI systems in line with patients’ concerns.

Despite the fact that patients have not interacted with robots, and are not aware of the functionalities and tasks that robots can actually perform in a hospital, robots are, for them, part of a possible healthcare scenario. AI and robotics shape the values and forms of governance of the present and future healthcare scenarios, although the effectivity and efficiency of AI and robotic systems for healthcare systems remain as a rhetorical claim [1]. From STS, interesting studies have been carried out on the representation of users accompanying robots [57]. These types of studies are based on the idea that throughout the technological design there is a process of inscription to embedding certain worldviews into artefacts [58]. Using this approach, our study shows another dimension of this sociotechnical assemblage: the process by which the worldviews and values inscribed in certain artefacts or technological systems are inscribed in citizens, shaping their values, symbolisms, concerns, etc. This process has also been identified in relation to welfare technologies, underlining the role of certain discourses and political proposals in the active construction of shared representations between citizens and technologies. Welfare technologies include a heterogeneous number of technologies and functions that are integrated in the four major types of application of robots proposed for their use in hospitals to manage a pandemic situation, such as communication support, assistive technologies, disease monitoring, remote treatment, entertainment, social and emotional support and stimulation, and help with everyday practical tasks [14]. Although citizens’ resistance and barriers to hinder these technologies are demonstrated, as well as resistance to their implementation by some municipal healthcare services [59], positive attitudes towards welfare technologies are growing [2]. This tendency is explained by the political discourse surrounding them, using the arguments for introducing welfare technologies embedded within the challenges of dealing with an ageing population and the shortage of care workers, the consequences of failing in the use of these technologies being increasing welfare costs and higher taxes for citizens, or a low quality of care for the individual care receiver [2].

In a similar vein, as is entangled in the responsible repertoire, automation in healthcare includes not only artefacts or systems with specific functionalities, appearances, or algorithms, but also technologies embedded in a political discourse that proposes robotics [60], AI, and ML as a solution for enhancing an efficient and effective healthcare future. In this sense, context-dependent repertoires are valuable tools to explore the relational boundaries between citizens and artefacts, to investigate the values, worldwide views, and political responses embedded in sociotechnical assemblages. Additionally, it is identified that social robots are key in the narrative of self-enhancement of older adults, thus reinforcing the idea of older adults that have to take care of themselves instead of shifting care responsibilities to the welfare state [57]; the patients’ responsible repertoire alerts us about the same process. This repertoire expresses clearly what Laval [61] calls the ‘dual nature’ of neoliberalism: it is not limited to the realm of state–market relations, but also refers to the articulation of governmental techniques of ‘behavioural conduction’. While the market in healthcare has been understood as innovation, and managerialism as efficiency [62], emphasis has been given to the promotion of responsible behaviour, individual choice, and autonomy by patients and citizens [63]. These results lead us to be wary of a dominant discourse in the media and academic literature about healthcare robotics, which narrows the range of available challenges and solutions to the problems that face the social organisation of care and healthcare systems [13]. Within the responsible repertoire, citizens assume an individual responsibility for healthcare, detaching it from welfare state and public responsibilities, positing the proper functioning of healthcare systems in the hands of the innovation market or private family management.

### 4.4. Aligning Care with Automation

In analysing patients’ perspectives on healthcare automation, we could see that there is no coherent or singular account. Concerns and opinions around robotics are not about stabilised artefacts, but about an ongoing process of collective discussion on how we want to care and be cared for. From citizens’ mobilisation of different interpretative repertoires, robots are virtual entities that could be actualised in many different ways. The notion of a robot acquires meaning through debates on how to organise care, these debates being part of robots’ heterogeneous and relational nature [64]. Thus, AI and robotic systems entail a discussion that is not focused on whether robots could or could not take care of us, or which tasks we could delegate to them, but about how we respond to the problems of care and healthcare systems. It is in this sense that AI, robotics, and ML could be considered a prime example of a political technology [65].

The repertoires of well-being and responsibility do not represent opposing or contradictory ideas or values, since both refer to the process of care, from different dimensions and political positions. As we show in the use of mobile phones during hospitalisation for COVID-19, technological artefacts are not independent devices, but elements that participate in an assemblage of care relations. As it appears in other studies, the narrative used by patients to explain their practices and relationships with digital health technologies (the mobile phone) does not move between binary notions such as affective/instrumental [5] or cold/warm [66]. In a situation of isolation and quarantine, the mobile phone makes possible the mediations that guarantee the patient’s care, virtually reconstructing, in the hospital, the network of social and emotional support relationships. In this way, the mobile phone becomes a particularly relevant node in the process of care during admission for COVID-19 (welfare repertoire). At the same time, this same mobile phone makes it possible for hospitalised citizens to follow up on their family members who are also ill with COVID-19, as well as making it easier for family members to organise their relationship with the hospital in order to follow up on the patient (responsibility repertoire). In bioethics, this is explained with relational theory, which highlights the need to analyse the role of technological artefacts in these sorts of assemblages or networks, for a better understanding of its social and ethical considerations [67]. The mobile phone does not care alone, it cares in a context of isolation measures to prevent the spread of the virus, at a time of oversaturation of the health system, in which a care assemblage is configured with relatives, friends, hospital infrastructures, etc. in which the mobile phone participates. Therefore, one of the main contributions of studying patients’ perspectives on future technologies, analysing their care experience in hospital, is that it emphasises the dependence of healthcare technologies on the political organisation and consideration of care, as well as what citizens think about them.

The example of the mobile phone shows how the integration of patients’ perspectives in the implementation of AI and robotics systems in hospitals has to rely on the possibility of using both interpretative repertoires to explain the same automation process. If we only focus on the accountability of citizens and the possible high-tech company proposals to increase efficiency and effectivity, robotics and AI for future healthcare will only reproduce existing dominant discourses and power relations, instead of helping to open the debates proposing disruptive models to the demographic challenge and the care crisis [13]. For example, when explaining their experience of hospitalisation for COVID-19, citizens were very dissatisfied with the way in which information about their health status was given to their relatives, who could not come to the hospital because of the confinement and quarantine measures. In some cases, the patient’s relatives were not given sufficient information on the evolution of their illness, while in others, clinical data on their evolution were not transmitted correctly. This type of episode caused discomfort among patients and relatives, anguish, and a feeling of mistrust and helplessness concerning the healthcare system. From the identification of this type of situation, robotic systems of communication between hospitalised patients and the outside world could be rethought to respond to the need to better manage and use data for ensuring an excellent follow-up for the relatives of that hospitalised patient. Thus, from the identification of a discomfort in the care experience and the demand for a more systematic and efficient form of relationship between the hospital and the relatives, the introduction of patients’ perspectives could offer valuable insights to politicians and designers and help align care to AI and robotic systems to meet the needs of citizens.

## 5. Conclusions

In this paper, we show how citizens’ care experiences could offer some insights on perspectives on AI and robotic systems in healthcare. Based on a set of interviews of citizens hospitalised during the first and second waves of COVID-19 in Catalonia, we identified that opinions and perceptions on automation could not be disassociated from a context of high pressures on the health system and a lack of resources, as well as from a political neoliberal discourse that governs high-income healthcare systems. From the research analysis, we found that, in line with other research on welfare technologies, patients’ perspectives on healthcare robots were ambivalent: on the one hand, they preferred to be cared for and to perform care practices with humans; and on the other, they considered that it could be very positive to introduce robots to take care of human beings, assisting carers and medical personnel and, if necessary, replacing them.

In contrast to explanations that account for the variability in citizens’ perspectives through the specification of care tasks that could be delegated to robots, assuming a fragmented notion of care and a relation between two independent entities (a human and a machine), we propose a more nuanced approach, embedded in a complex comprehension of social action and technoscientific assemblages of care. Using the notion of interpretative repertoires [54], we identify how citizens’ apparently contradictory perspectives on healthcare robots, when analysed closely, account for different dimensions of care. Thus, we identify two interpretative repertoires that are mobilised in citizens’ perspectives on robotics: a well-being repertoire, associated with human-to-human relations and the notion of ‘good care’, and a responsibility repertoire, associated with individual and collective accountability to facilitate the proper functioning of the system and the guarantee of health assistance in a context of healthcare pressure. From citizens’ mobilisation of different interpretative repertoires, robots are virtual entities that acquire different meanings through the debates around them; thus, healthcare robotics entail a political discussion that materialises the care crisis. In this way, analysing patients’ perspectives allows us to move beyond the dominant discourse of innovative technology markets to respond to healthcare challenges.

Entangled in a responsible repertoire, in a political neoliberal response to the care crisis, citizens assume an individual responsibility for health care, detaching it from welfare state and public responsibilities, positing the proper functioning of healthcare systems in the hands of the innovation market or private family management. To offer valuable insights to politicians and designers to serve citizen’s needs, the integration of patients’ perspectives on the implementation of AI and robotics systems in hospitals has to rely on the possibility of using the well-being and the responsibility interpretative repertoires to explain the same automation process. Both are relevant in order to grasp the complexity of patients’ approaches to automatisation of health care. Although we do not expect our study to diminish the rhetorical debate around robotics and AI in health care, we hope that our analysis can contribute to underline the need to place the concerns, values, and opinions of citizens at the centre of the development and implementation processes of AI and robotics systems in health care, as the only way to ensure that these technologies seek to respond to individual and collective well-being and good living.
